# Procedures to combine estimators of greenhouse gases emission factors

**DOI:** 10.1186/s13021-024-00250-8

**Published:** 2024-02-05

**Authors:** Ernesto C. Marujo, Gleice G. Rodrigues, Arthur A. Covatti

**Affiliations:** 1 Department of Fundamental Sciences, Instituto Tecnológico de Aeronáutica, S.J. dos Campos, SP Brazil; 2 Deep ESG, São José dos Campos, SP Brazil

**Keywords:** Emission factors combination, Emissions uncertainties, Meta-analysis for emission factors, Pooling emission factors

## Abstract

**Background:**

This article describes a new procedure to estimate the mean and variance of greenhouse gases (GHG) emission factors based on different, possibly conflicting, estimates for these emission factors. The procedure uses common information such as mean and standard deviation usually reported in IPCC (Intergovernmental Panel on Climate Change) database and other references in the literature that estimate emission factors. Essentially, it is a procedure in the class of meta-analysis, based on the computation of ${S}_{a}^{2}$, a new estimator for the variance of the emission factor.

**Results:**

We discuss the quality of this estimator in terms of its probability distribution and show that it is unbiased. The resulting confidence interval for the mean emission factor is tighter than those that would have resulted from using other estimators such as pooled variance and thus, the new procedure improves the accuracy in estimating GHG emissions.

The application of the procedure is illustrated in a case study involving the estimation of methane emissions from rice cultivation.

**Conclusions:**

The estimation of emission factors using ${S}_{a}^{2}$ was demonstrated to be more accurate because it is not biased and more precise than alternative methods.

**Supplementary Information:**

The online version contains supplementary material available at 10.1186/s13021-024-00250-8.

## Background

The bottom-up method to estimate the emission of GHG (greenhouse gases) of a process consists of measuring (*A*), the amount of an activity or material used during a time period, and multiplying this quantity by (*F*), the specific emission factor of that activity or material [3, 9, 38].

In agriculture, mining and many other economic activities, the uncertainty of both factors, *A* and *F*, are important and the variance of the product is calculated using the error propagation formula, IPCC [18]. More importantly, if both factors are correlated, the expected value of the product is not simply the product of the expected values [29, 30]. See the Additional file Material for a summary of important concepts involved in the propagation of uncertainty.

Mukhigulishvili et al. [31], argue that, in the context of estimating the emissions associated with activities of a company plant, the uncertainty about *A* may be small and it is reasonable to assume that *A* is known precisely and is not subject to uncertainty or random variations. Then, the possible variation in the volume of GHG emitted is attributed to uncertainties related to *F*.

Emission factors are reported in platforms, such as IPCC [19] that compile and publish experimental results that follow specific protocols to guarantee the homogeneity of methods and criteria. However, these factors may not be applicable in certain countries or regions due to unique conditions that are not always represented in the IPCC database [32].

Emission factors reflect the mean emission rate obtained from a set of available data, [10]. Therefore, it may not be a trivial task to verify if a tabulated emission factor is applicable to a specific situation [22]. Kono et al. [23] observed potential underestimations and overestimations of GHG emissions in the German electricity grid which ranged from + 22% (October 2015 weeknights) to − 34% (May 2015 weekend daytime).

It is not unusual to observe emission factors differing strikingly over 50%, depending on who evaluates it, the conditions at the time of emission measurement, and other causes [40]. Pouliot et al. [35] analyzed a compilation of air pollutant emission factors for combustion-based NO_x_ emissions, and they showed that, for a specific year, more than half of the emission factor values had not been updated with current data and that the quantitative uncertainty ranges were between 25 and 62%. According to Verma et al. [41] and Doiron et al. [8], an appropriate combination of estimates using secondary data called pooling of estimates (in contrast with pooling of data, which would be an aggregation of micro-level data) is a valuable tool to enhance the statistical power required to investigate relatively rare phenomena.

As Leito et al. [27] demonstrated, an important issue in combining the results of different studies is the variance in the estimators. This becomes particularly evident when dealing with emission estimators as Fajgelj et al. [11] show. Even when employing the most advanced techniques and rigorous data collection procedures, emissions associated with an activity are inevitably subject to uncertainty, due to numerous potential statistical disturbances. Consequently, different research groups may yield disparate results in estimating emission factors.

This work aims to investigate procedures to combine information about emission factors to produce the most accurate estimate for the emission factor of an activity. The task is relevant because identifying or estimating the correct emission factor for an activity is crucial for the reliability of emission estimation results. The task is also non-trivial because in many practical instances, as shown in Whitaker et al. [43], emission factor estimates are subject to variance and uncertainty and may show rather different mean values that need to be combined.

As a case study, we considered the agriculture of rice in Central Vietnam and tested possible estimates for its GHG emission. In that case, there were three possible estimates for the emission factor of CH_4_. The application of the new procedure presented in this paper resulted at an estimate of the emission factor that was unbiased and of least variance, therefore, most accurate among several other methods.

### Previous works

In 2006, several international agencies (APAT, IUPAC, BIPM, IAEA, ISO and UNIDO) organized a workshop to discuss the issue of combining analytical results [11]. As a result, they stated that analytical laboratories, working independently, using different analytical methods or, more likely, collaborative analysis, produce robust mean and robust standard deviation for each set of analytical results and that the “assigned values are then determined as the robust average of all laboratories mean values, while the expanded uncertainty range is calculated as reported in ISO 13528”.

Fajgelj et al. [11] presented a review of the theoretical grounds for combining statistical results recognizing that the work of Cochran [6] had established the fundamentals for studies in this theme. The authors examined first the question of how to form an average of measurements considering only linear averages, called weighted means, and discussed reasons to adopt other possible weights.

The theme of combining statistical results receives different names, according to the context of the application. For statisticians, it may be referred to as meta-analysis. In the field of chemistry or physics, it is studied under the umbrella of interlaboratory studies; in human sciences, combining evidence. As an example of the application of this last category, Juchli [21] investigated the problem of combining different pieces of evidence to form a consensus in the context of forensic judgments.

In Kulinskaya et al. [24] the authors discuss several techniques used to combine comparable studies in order to obtain a more precise estimate of an effect. Perhaps the most important lesson from the meta-analysis literature is that “if we combine measurements using weights that are inversely proportional to the variance of the measurements, the weighted average is an efficient estimator of the measurand.” [25]. This is not hard to show using partial derivatives, as shown by Rabinovich [36]. Therefore, Eq. (1) and Eq. (2) present the unique minimum variance unbiased estimator (UMVUE) of *μ* under the normality assumption and the best linear unbiased estimator (BLUE) even without normality.

1 $$\widehat{\mu }=\frac{{\sum }_{i=1}^{k}{w}_{i}\overline{{f }_{i}} }{{\sum }_{i=1}^{k}{w}_{i}}$$

2 $${w}_{i}=\frac{{n}_{i}}{{\sigma }_{i}^{2}}$$

where $$\overline{{f }_{i}}=\frac{1}{{n}_{i}}{\sum }_{i=1}^{{n}_{i}}{f}_{ij} .$$

There is, however, a practical problem: the true value of each study variance ${\sigma }_{i}^{2}$ is typically unknown and we have to recur to their estimates ${S}_{i}^{2}$. The problem becomes more intricate and there is no closed form solution for determining $\widehat{\mu }$, since we would have to use ${S}_{i}^{2}$ to compute $\widehat{\mu }$ but the computation of those also depends on $\widehat{\mu }$.

Bartlett and Frost [2] and, more recently, Huang [15] studied methods to determine the consensus of laboratory studies. The methods use approximations to estimate variances. Some of these methods require iterative procedures.

Hahn and Raghunathan [13] proposed a Bayesian procedure that, from previous distributions and from new data, they determined the posterior probability distributions for the estimate of the population mean. If one considers that all data represent previous information and that there is no conditioning event (representing new data), one could show that Bayes’ theory would result in a simple weighing method, as presented before.

From the literature review, one may conclude that the point estimation of the mean emission factor, using a combination of different estimates is well solved for the most relevant cases. Nevertheless, determining an interval estimation for this mean depends on the characterization of the variance and distribution of the point estimator. That is the issue focused on the following Sections.

### Absolute or relative variation

The use of absolute or relative standard deviations will depend on the characteristic of the random variable representing the emission factor *F*. If we could model *F* as a normal random variable, then we should use absolute standard deviations. If *F* is better modeled as a log-normal random variable, then it is better to use relative variations. In this case, the expression of *F* in relative terms, usually in percentages, will be a normal random variable.

Olofsson [34] suggested that in some contexts, it is convenient to combine or pool sample variances not in absolute but in relative (or percentage) terms.

In the following developments, we consider that *F* can be appropriately modeled as a normal random variable, although the development could be easily accommodated for the case of log-normal distribution. IPCC [18] suggests that, unless there is clear evidence to the contrary, the probability density function of emission factors should be assumed to be normal.

## Methods

### Point estimators for the mean emission factor μ

If we have *k* independent samples of the same population and each sample contains ${n}_{i}$ elements, then each sample mean $\overline{{f }_{i}}$ is an unbiased estimator of *μ.* Combining the information in all available samples will provide another estimator of *μ*, the overall average, $\overline{\overline{f}}$ (Eq. (3)) that is also an unbiased and efficient estimator of *μ*:

3 $$\widehat{\mu }=\overline{\overline{f}}=\frac{1}{{\sum }_{i=1}^{k}{n}_{i}}{\sum }_{i=1}^{k}{n}_{i}\overline{{f }_{i}}$$

Other estimators, using the median of ${f}_{ij}$, for example, would work better if the data contains outliers. Nevertheless, under the assumption of normality of* F*, we can say that $\overline{\overline{f}}$ is an estimator of good quality in the sense that this estimator is unbiased, has the smallest variance and, therefore, would enable us to construct a tight confidence interval for *μ*.

However, the computation of $\overline{\overline{f}}$ using Eq. (3) may not be possible, for instance, if we do not know the number of elements in each sample. In such cases, an ad hoc procedure would have to be used. For instance, we could consider all sample sizes ${n}_{i}$ to be equal to each other. That assumption, of course, would be questionable if there is evidence to the contrary. According to Oliveira [26, 28, 33] sample sizes for determining CO_2_ emission factors are in the range of 10 to 41.

Another issue to consider is the assumption of independence. We must recognize that in many circumstances the assumption of independent samples taken randomly from the population may be violated. Nevertheless, we propose to continue using this statistically friendly assumption in the hope of producing useful conclusions and insights for this case and subsequently, investigating special methods for when this assumption is violated.

We advocate in favor estimating *μ* using $\overline{\overline{f}}$, which corresponds to adopting weights ${w}_{i}={n}_{i}$ instead of the ones suggested in Eq. (2). The reason is that if we do not know ${\sigma }_{i}^{2}$ we must recourse to approximations and these approximations render the resulting estimator of a random variable that is difficult to characterize. Two classical approximation methods are the Grabill and Dean (GD) and the Mandel and Paule (MP) procedures (See Additional file 1: Material).

The GD estimator of $\mu$, $\widehat{{\mu }^{GD}}$ has a variance that depends on expectations and variances of ${S}_{i}^{2}$ and, therefore, $\widehat{{\mu }^{GD}}$ is not easy to determine [14] and require approximate and iterative procedures. Similarly, using the MP estimator would require iterative procedures and the distribution of the estimator is virtually impossible to determine.

We shall circumvent such difficulties of GD or MP estimators by using weights ${w}_{i}={n}_{i}$ and, thus arrive at $\widehat{\mu }$ as in Eq. (3).

Another reason to prefer $\overline{\overline{f}}$ to estimate $\widehat{\mu }$ is particularly relevant in the context of combining results for measurements of GHG emissions. In such context, the inverse of the sample variance may not be a good measure of the accuracy of the *i*^th^ result. Our argument is of a practical nature: When estimating GHG emissions, sample variances sometimes are the result of expert opinions and models, not exactly experimental values. Moreover, sample variance data might include outliers. If a sample variance is erroneously reported as close to zero, the weighting average of Eq. (2) will be far from the true mean. Therefore, we argue that it is justifiable to use the overall mean $\overline{\overline{f}}$ of Eq. (3) to form a point estimate of the emission factor.

### Interval estimators for μ

Once we produce a point estimator for the mean emission factor, it is natural that we investigate the quality of that estimator. One important measure of the quality of an estimator is its possible bias. Under the common assumptions of independence of samples and homogeneity of the population, there would be no bias. Where these assumptions do not apply, one might need to investigate possible bias considering the specificities of the case.

Other two important measurements of the quality of an estimator are its variability and its confidence interval. The construction of a proper confidence interval for a parameter depends not only on determining the variance of the estimator but also on its distribution profile.

If $\overline{\overline{f}}$ is the average of a normally distributed random variable of mean *μ* and variance ${\sigma }^{2}$ and if ${S}^{2}$ is an estimator for ${\sigma }^{2}$ computed according to Eq. (4), then, for $N=\left({\sum }_{i=1}^{k}{n}_{i}\right),$ the ratio $(N-1){S}^{2}/{\sigma }^{2}$ has probability distribution Chi-square with $(N-1)$ degrees of freedom. Therefore, $T=(\overline{\overline{f}}-\mu )/(S/\surd N)$, is a standardized variable distributed as a Student’s T with $(N-1)$ degrees of freedom [5].

4 $${S}^{2}=\frac{1}{({\sum }_{i=1}^{k}{n}_{i})-1}\left({\sum }_{i=1}^{k}{\sum }_{j=1}^{{n}_{i}}{({f}_{ij}-\overline{\overline{f}})}^{2}\right)$$

Consequently, the confidence interval for the true mean value of* F* is formed using critical values, ${t}_{\left(N-1\right),\frac{\alpha }{2}},$ of the Student’s T random variable as in Eq. (5).

5 $$\overline{\overline{f}} - t_{{\left( {{N - 1} } \right)}} ,_{\frac{\alpha}{2}} \frac{s}{\sqrt N } \le \mu \le \overline{\overline{f}} + t_{{(n - 1),{\frac{\alpha}{2}} }} \frac{s}{\sqrt N }$$

### Estimators for the variance of the emission factor *σ*^*2*^

There are many possible estimators for the true variance ${\sigma }^{2}$. We will present two classic estimators and propose a third one, called ${S}_{a}^{2}$.

In the following paragraphs, we will present a summary for the properties of estimators ${S}_{p}^{2}$ and ${nS}_{m}^{2}$. The Additional file Material contains the details of the derivations of these properties.

### Pooled variation estimator ${{\varvec{S}}}_{{\varvec{p}}}^{2}$

Under certain circumstances, the estimation of the variance of* F* could use a procedure called pooled variance. The technique is applicable if we believe that there are unforeseen variations in the mean, but not in the variance of the emission factor from one sample to another. For example, if ${f}_{ij}$ is modeled by a function of an explanatory variable ${x}_{i}$ and a random component ${\varepsilon }_{ij}$: ${f}_{ij} = a {x}_{i}+{\varepsilon }_{ij}$. Then, if we can estimate $a$ and have control on the value of ${x}_{i}$, the only random variation is embedded in ${\varepsilon }_{ij}$ and our interest is the estimation of the variance of ${\varepsilon }_{ij}$.

From each sample, we could find an estimator for the population variance using ${S}_{i}^{2}$ defined Eq. (4). If ${f}_{ij}$ are independent observations of random variable *F*, it is easy to show that ${S}_{i}^{2}$ is an unbiased estimator of ${\sigma }^{2}$ [5].

We are interested in estimating the variance of a population based on a pool of samples. In this case, the literature suggests that the variance of the population can be estimated by the pooled variation ${S}_{p}^{2}$ (Eq. 6) [20].

6 $${S}_{p}^{2}=\frac{1}{{\sum }_{i=1}^{k}({n}_{i}-1)}{\sum }_{i=1}^{k}{({n}_{i}-1)S}_{i}^{2}$$

Following Cochrane’s theorem [39], it is possible to verify that, under the condition that the emission factor* F* is a normally distributed random variable with variance ${\sigma }^{2}$, then ${S}_{i}^{2}$ would follow a scaled chi-square distribution with $\left({n}_{i}-1\right)$ degrees of freedom [5] 1

Three conditions are necessary to characterize ${S}_{p}^{2}$ precisely as a scaled chi-square random variable with ${df}_{p}={\sum }_{i=1}^{k}\left({n}_{i}-1\right)$ degrees of freedom. We refer to these conditions as the “independence, normality and homogeneity” criteria: (i) independent simple random samples; (ii) normally distributed populations and (iii) equal population variances [1, 4].

Assuming that all samples are independent, or simply not correlated, it can be shown that ${S}_{p}^{2}$ is unbiased and is the most efficient estimator in the form of linear combinations of the sample variances [7].

Thus, we can construct the confidence interval for ${\sigma }^{2}$. This confidence interval would be the non-symmetrical interval in Eq. (7).

7 $$\frac{{df}_{p} {S}_{p}^{2}}{{\chi }_{{1-\frac{\alpha }{2}; df}_{p} }^{2}}\le {\sigma }^{2}\le \frac{{df}_{p}{ S}_{p}^{2}}{{\chi }_{{\frac{\alpha }{2}; df}_{p} }^{2}}$$

where ${S}_{p}^{2}$ is the point estimator for ${\sigma }^{2}$, ${df}_{p}={\sum }_{i=1}^{k}\left({n}_{i}-1\right)$ and ${\chi }_{{1-\frac{\alpha }{2}; df}_{p} }^{2}$ and ${\chi }_{{\frac{\alpha }{2}; df}_{p} }^{2}$ are the critical values of a chi-square distribution with appropriate degrees of freedom.

The variance of ${S}_{p}^{2}$ is given by Eq. (8).

8 $$Var\left({S}_{p}^{2}\right)=\frac{2{\sigma }^{4}}{{\sum }_{i=1}^{k}\left({n}_{i}-1\right)}$$

The population* F*, however, may not be normally distributed. In this case, the point estimates for *μ* and *σ*^2^ are still valid but the distributions of their estimates no longer follow, respectively, Student’s T and chi-square distributions.

### Variance estimator ${({\varvec{n}} {\varvec{S}}}_{{\varvec{m}}}^{2})$: using sample’s means only

In this section, we will overview the possibility of estimating ${\sigma }^{2},$ the variance of the population of random variable *F* if we only know the value of the averages of a set of samples.

Consider that the number of elements in each sample is ${n}_{i}$ and that ${n}_{i}$ equals *n* for all *i*. Let us call $\overline{F }$ the random variable representing the average of *n* random variables *F.* Since* F* is supposed to be a normal random variable, $\overline{F }$ is also a normal random variable with variance equal to ${\sigma }^{2}/n$ and, thus, ${\sigma }^{2}= nVar\left(\overline{F }\right)$.

Observe that $Var\left(\overline{F }\right)$ can be estimated by the sample variance of $\overline{F }$*,* here called ${S}_{m}^{2}$, that is computed from a sample of elements, $\overline{{f }_{1}},\overline{{f }_{2}},\dots ,\overline{{f }_{k}}$ as in Eq. (9).

9 $${S}_{m}^{2}=\frac{1}{(k-1)}{\sum }_{i=1}^{k}{(\overline{{f }_{i}}-\overline{\overline{f}})}^{2}$$

Therefore, we produced another unbiased estimator for $${\sigma }^{2}$$, the product ($${n S}_{m}^{2})$$.

${S}_{m}^{2}$ can be recognized to be a scaled chi-square random variable 2 with ${df}_{m}=\left(k-1\right)$ degrees of freedom.

In Kulinskaya et al. [24], one could also confirm that, under the normal model, the between group sum of squares is distributed as a chi-square random variable. Although ($${n S}_{m}^{2})$$ is unbiased and is the simplest estimator of $${\sigma }^{2}$$ to compute, it cannot be computed if we do not know *n.* In fact, if we have all values of $$\overline{{f }_{i}}$$*,* but we do not know the value of *n*, we could not find bounds or limits to ($${n S}_{m}^{2})$$ since it increases linearly with *n*.

The precision of ${(n S}_{m}^{2})$ as an estimator of *σ*^2^ may be evaluated by its variance. After some simple algebraic developments presented in Additional file Material, such variance is given in Eq. (10).

10 $$Var\left(n{S}_{m}^{2}\right)=\frac{{2\sigma }^{4}}{k-1}$$

Observe that $Var\left({nS}_{m}^{2}\right)$ is considerably higher than $Var\left({S}_{p}^{2}\right)$ for any $n$ greater than two.

We will now define ${S}_{a}^{2}$ and show that it has better properties than ${S}_{p}^{2}$ and $\left({nS}_{m}^{2}\right)$.

### Variance estimator ${{\varvec{S}}}_{{\varvec{a}}}^{2}$

The estimator ${S}_{a}^{2}$ is meant to be applicable when the variation within each group and the variation intergroups are both relevant to estimating the population variance. The idea of this estimator ${S}_{a}^{2}$, is to use a combination of previous estimators ${S}_{p}^{2}$ and ${(nS}_{m}^{2})$, thus, considering both sources of variations.

We define ${S}_{a}^{2}$ using the weighted average of ${S}_{p}^{2}$ and ${(nS}_{m}^{2})$ as in Eq. (11), with specific values of *w*_1_ and *w*_2_ that we will define conveniently.

11 $${{S}_{a}^{2}={w}_{1}S}_{p}^{2}+{{w}_{2}nS}_{m}^{2}$$

In our model, we suppose that there are* k* research groups that estimated the mean and variance of *F.* Each group might work with a subset of the population and arrive at a different estimate for the mean and variance of what we assume to be a homogeneous population* F*.

We shall consider that the only reason for different means in each group is the existence of variance in the population. In order to mark this condition, we state Assumption 1, that we use throughout the following sections.

*Assumption 1* there is a unique population* F* with a unique mean *μ* and unique variance *σ*^2^.

The estimation of *σ*^2^ and its distribution is our immediate goal and we shall use a procedure based on ANOVA and the total variance formula [36].

### Probability distribution of estimators of intragroup and intergroups variances

We have determined that ${S}_{p}^{2}$, the pooled variance estimator is a scaled chi-square random variable because ${S}_{p}^{2}$ is linked to the chi-square random variable ${C}_{p}^{2}$ with ${df}_{p}$ degrees of freedom.

In the second part of the ${S}_{a}^{2}$ formula, $\left({nS}_{m}^{2}\right)$ is also linked to a chi-square random variable with ${df}_{m}$ degrees of freedom. In this case, ${C}_{m}^{2}$.

However, the weighted sum of ${C}_{p}^{2}$ and ${C}_{m}^{2},$ two chi-square distributed random variables, is not necessarily a chi-square distributed random variable.

This observation is of major importance since it precluded us from using a well-known theorem of Statistics to establish the functional form of the probability distribution of ${S}_{a}^{2}$.

The combination of chi-squared variables is a challenge that Ferrari [12] proposed to solve using approximate expressions. The problem is especially intricate when the variables involved are correlated. In our case the possible correlation between ${C}_{p}^{2}$ and ${C}_{m}^{2}$ would have to be investigated and then, approximate formulas would have to be used to characterize the distribution of the sum ${{w}_{1}S}_{p}^{2}+{{w}_{2}nS}_{m}^{2}$.

The key to resolve this challenge is to define weights *w*_1_ and *w*_2_ conveniently, rendering meaningful ${S}_{a}^{2}$ and providing ${S}_{a}^{2}$ with statistical properties that would allow us to determine its probability distribution without determining the correlation between ${C}_{p}^{2}$ and ${C}_{m}^{2}$.

The definition of the weights we propose in this paper is the result of an analysis of the degrees of freedom of the statistics involved and is inspired by the success of the development of the formula of pooled variance.

The resulting weights are simple and compatible with ANOVA procedures used in classical tests of differences of means. Also, it presents what we think is a remarkable property: they produce an estimate of total variability that combines intragroup variability estimates with intergroups variability estimates to produce a statistic ${S}_{a}^{2}$ that is a scaled chi-square distribution. The guarantee that ${S}_{a}^{2}$ is a scaled chi-square distribution is essential to determine confidence intervals for the true total variance.

### Formula for ${{\varvec{S}}}_{{\varvec{a}}}^{2}$ and its distribution

We define ${w}_{1}$ and ${w}_{2}$ by expressions Eqs. (12), (13).

12 $${w}_{1}=\frac{{\sum }_{i=1}^{k}\left({n}_{i}-1\right)}{{\sum }_{i=1}^{k}\left({n}_{i}\right)-1}$$

13 $${w}_{2}=\frac{k-1}{{\sum }_{i=1}^{k}\left({n}_{i}\right)-1}$$

In the following derivations, we will use the familiar notation of “sum of squares”: SST, SSW and SSB:

$SST = \sum\limits_{i = 1}^{k} {\sum\limits_{j = 1}^{ni} {\left( {f_{ij} - \overline{\overline{f}} } \right)^{2} } }$: representing the total sum of squared deviations from each observation to the global sample mean

$SSW = \sum\limits_{i = 1}^{k} {\sum\limits_{j = 1}^{ni} {\left( {f_{ij} - \underline{fi} } \right)^{2} } }$: representing the sum of the variations within each group

$SSB = \sum\nolimits_{i = 1}^{k} {ni} \left( {\underline{{f_{i} }} - \overline{\overline{f}} } \right)^{2}$: representing the intergroups or, between groups variation.

Recall that, using the “law of total variance”, we have: SST = SSW + SSB. If we expand the formula for ${S}_{a}^{2}$ of Eq. (15), we get equations Eq. (18) to Eq. (21).

14 $$S_{a}^{2} = w_{1} \frac{{\sum\nolimits_{i = 1}^{k} {\sum\nolimits_{j = 1}^{ni} {\left( {f_{ij} - \underline{{f_{i} }} } \right)^{2} } } }}{{\sum\nolimits_{i = 1}^{k} {\left( {n_{i = 1} } \right)} }} + w_{2} \frac{{\sum\nolimits_{i = 1}^{k} {\sum\nolimits_{j = 1}^{ni} {\left( {\underline{{f_{i} }} - \overline{\overline{f}} } \right)^{2} } } }}{k - 1}$$

15 $${S}_{a}^{2}={w}_{1}\frac{SSW}{{\sum }_{i=1}^{k}({n}_{i}-1)}+{w}_{2}\frac{SSB}{(k-1)}$$

16 $${S}_{a}^{2}=\frac{SSW}{{\sum }_{i=1}^{k}({n}_{i})-1}+\frac{SSB}{{\sum }_{i=1}^{k}({n}_{i})-1}$$

17 $${S}_{a}^{2}=\frac{SST}{{\sum }_{i=1}^{k}({n}_{i})-1}$$

Therefore, if we use the proposed weights ${w}_{1}$ and ${w}_{2}$, we arrive at a very simple expression for ${S}_{a}^{2}.$ From this expression of ${S}_{a}^{2}$, it is also easy to determine its probability distribution: following Cochrane´s theorem, if* F* is a normal random variable, then ${S}_{a}^{2}$ is made of a sum of squared standard normal variables and, thus, ${C}_{a}^{2}$ defined as in Eq. (18), is distributed as a chi-square random variable with ${df}_{a}$ degrees of freedom (Eq. 19) [1].

18 $${C}_{a}^{2}=\frac{\left[\left({\sum }_{i=1}^{k}{n}_{i}\right)-1\right]{S}_{a}^{2}}{{\sigma }^{2}}$$

19 $${df}_{a}=\left({\sum }_{i=1}^{k}{n}_{i}\right)-1$$

${S}_{a}^{2}$ is proven to be an unbiased estimator for *σ*^2^ for the usual condition of normality, homogeneity, and independence of samples [7]. It is important to note that we have shown that ${C}_{a}^{2}$ is a chi-square random variable even though we have not shown the non-correlation between ${S}_{p}^{2}$ and ${(nS}_{m}^{2}).$

### Computation of ${{\varvec{S}}}_{{\varvec{a}}}^{2}$

In Eq. (17), the formula proposed for ${S}_{a}^{2}$, the summation SST involves information about each measurement ${f}_{ij}$. If we had information regarding each ${f}_{ij}$ we could also use stratified sampling, bootstrapping, or other resampling methods to improve the quality of the estimator for the emission factor. However, this information is seldom available in the IPCC database or in the literature where experimental results for emission factors are published.

This is the point where the estimator ${S}_{a}^{2}$ is advantageous: if we use the formula of Eq. (11), ${{S}_{a}^{2}={w}_{1}S}_{p}^{2}+{{w}_{2}nS}_{m}^{2}$, we can easily compute ${S}_{a}^{2}$ since the values involved are usually available.

### Precision of estimator ${{\varvec{S}}}_{{\varvec{a}}}^{2}$

The precision of ${S}_{a}^{2}$ as an estimator of *σ*^2^ may be evaluated by its variance. The variance of ${S}_{a}^{2}$ is determined by recognizing that ${Var(C}_{a}^{2})=2{df}_{a}$ and is written as in Eq. (20). The confidence interval for the true parameter *σ*^2^ is expressed as in Eq. (21).

20 $$Var\left({S}_{a}^{2}\right)=\frac{2{\sigma }^{4}}{\left({\sum }_{i=1}^{k}{n}_{i}\right)-1}$$

21 $$\frac{{df}_{a} {S}_{a}^{2}}{{\chi }_{1-\frac{\alpha }{2};{df}_{a}}^{2}}\le {\sigma }^{2}\le \frac{{df}_{a}{ S}_{a}^{2}}{{\chi }_{\frac{\alpha }{2};{df}_{a}}^{2}}$$

where:

${\chi }_{\frac{\alpha }{2};{df}_{a}}^{2}=$ Inverse cumulative distribution, for probability equal to α/2, of a chi-square distribution with ${df}_{a}$ degrees of freedom,

${\chi }_{1-\frac{\alpha }{2};{df}_{a}}^{2}=$ Inverse cumulative distribution, for probability equal to (1-α/2), of a chi-square distribution with ${df}_{a}$ degrees of freedom.

Observe that $Var\left({S}_{a}^{2}\right)$ is smaller than $Var\left({S}_{p}^{2}\right)$ that, in turn, is smaller than $Var\left({nS}_{m}^{2}\right)$.

## Results

### Simulations to confirm the quality of the estimators of ***σ***^2^

Each of the three methods previously presented provide an estimator for *σ*^2^. Depending on the estimator for *σ*^2^ and its probability distribution, we can draw a confidence interval for *μ*.

The method of ${S}_{a}^{2}$, based on the law of total sum of squares, requires more information but provides estimates that do not neglect the intergroups variation (disregarded in the ${S}_{p}^{2}$ method) nor neglect the intragroup variation (disregarded in the $({nS}_{m}^{2})$ method).

Because each method uses different pieces of data, it is not clear how to compare their quality. Therefore, we use a simulation exercise to illustrate the differences in procedures and results of the three methods. We consider a population of observations of a normal random variable* F* with zero mean and variance equal to 1. In each simulation run, we generate 20 pseudo-random numbers and divide them into 4 groups of equal size *n* = 5 each. We then compute the sample’s means, sample’s variances and the estimators for the variance ${S}_{p}^{2}$, ${S}_{a}^{2}$, and ${nS}_{m}^{2}$. For the computation of ${nS}_{m}^{2}$, even though the value of *n* would not be known, we used *n* = 5.

The point estimates of *μ* are the same for the three methods and were obtained using (Eq. 7). We run 1000 simulations. The average estimates for *σ*^2^ using ${S}_{p}^{2}$, ${S}_{a}^{2}$, and ${nS}_{m}^{2}$ were, respectively: 1.0017; 1.0055 and 1.0258. These values seem very close to one another and confirm the unbiased characteristic of the three estimators. However, the qualities of these estimators are different. The probability distribution of the three estimators of *σ*^2^ are scaled chi-squares, but with different degrees of freedom, implying different variances in these estimators.

For the example with *σ*^2^ = 1; ${n}_{i}=n=5$ and $k=4$, we computed ${Var(nS}_{m}^{2})=2/3$, much higher than $Var\left({S}_{p}^{2}\right)=2/16$ which is also a little higher than $Var\left({S}_{a}^{2}\right)=2/19$. The simulation results confirmed that ${S}_{a}^{2}$ has the smallest variance of all. The pooled variance ${S}_{p}^{2}$ has an intermediate value of variance and $({nS}_{m}^{2})$ has the highest variance. Therefore, when comparing these methods, we consider ${S}_{a}^{2}$ to be the preferable estimator for *σ*^2^. The three estimators are unbiased, but ${S}_{a}^{2}$ has a smaller variance (Eq. (22)).

22 $$Var\left({S}_{a}^{2}\right)\le Var\left({S}_{p}^{2}\right)\le Var\left({nS}_{m}^{2}\right)$$

Since $\left({nS}_{m}^{2}\right)$ has the largest variance, we consider it the least preferred method.

**Fig. 1:**
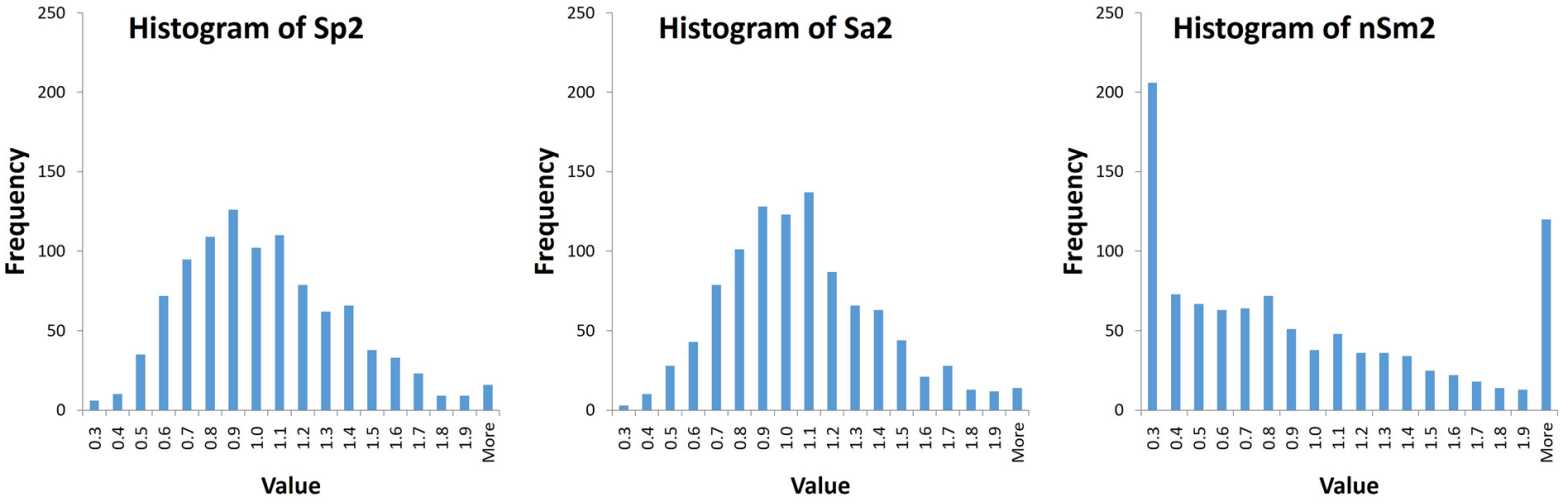
Histograms of estimates of σ^2^ in 1000 simulations of **a** ${S}_{p}^{2}$; **b** ${S}_{a}^{2}$; **c** ${nS}_{m}^{2}$

Figure 1 shows the histograms of the point estimates of *σ*^2^ obtained by the three methods in the simulations. The histograms confirm the theoretical conclusion that the variability of ${S}_{a}^{2}$ was the smallest.

Observe that Table 1 shows that the averages of all estimators were close to the real parameter *σ*^2^ and the variance of these estimators in the simulations were in the order we expected, with the least variation for ${S}_{a}^{2}.$

**Table 1:** Estimates for the variance in 1000 simulations

Estimators	${S}_{p}^{2}$	${S}_{a}^{2}$	${nS}_{m}^{2}$
Average	1.0017	1.0055	1.0258
Standard deviation	0.363	0.345	0.867

We must remember that we used the right value of *n* in the computation of ${nS}_{m}^{2}$. In practice, the value of *n* would not be known, and an arbitrary choice of* n* could greatly alter the results. Even in the event of chance where we choose the right* n*, the estimated variance of ${(nS}_{m}^{2}),$ confirmed in the histograms for the simulations, shows that it is spread over a large interval and therefore, an instance of such an estimator could be far off the real value of *σ*^2^. This means that $({nS}_{m}^{2})$ should be used only when there is no alternative.

## Case study

We considered the study of Vo et al. [42]. They analyzed the methane emission factors from Vietnamese rice production in flooded fields. One of their conclusions was that the season is more important than the edapho-hydrological characteristics of the zones for explaining differences in emission factors.

From that study we collected the statistics relative to means and standard deviations of observations of methane emissions in several field sites and cropping seasons (early, mid and late-year seasons).

The emission factors were developed from field measurements using the closed chamber technique. The fluxes of CH_4_ (and N_2_O) were determined using the static flux chamber technique and gas chromatographic analyses of gas samples. Sampling was conducted on average with three replicates, 10 sampling dates per season and four gas samples per chamber exposure. The analysis followed the IPCC Tier 2 methodology [16, 17]. The resulting CH_4_ emission factors are presented in Table 2. The first columns refer to emissions per area per day. In order to get the emission factor in terms of kg of methane per ton of rice produced, we have only to multiply by the number of days of the cropping time and divide by the yield in terms of tons per ha.

**Table 2:** Emission of methane in the production of rice at specific sites in Vietnam in 2018

Site	Emission of CH_4_		Period (days)	Yield (ton ha^−1^)	Emission of CH_4_	
	*(kg ha*^*−1*^* day*^*−1*^*)*				(kg ton^−1^)	
	Mean	St Dev			Mean	St. Dev
C2	1.444	0.058	109	7.6	20.7	0.83
C3	1.948	0.019	110	7.5	28.6	0.28
C4	1.853	0.088	108	7.3	27.4	1.30

Source: Vo et al. [42]

We selected the sites named C2, C3 and C4 which are very close to each other in central Vietnam, near the city of Huê. The data involved the same crop season of early 2018.

The computations considered sample sizes all equal to $n=10$ and number of samples $k=3$. We computed the weighted average of the sample means to estimate the overall mean emission factor. The weights were all equal to be consistent with the computation of $\overline{\overline{f}}$ and the result was 25.6 using Eq. (3).

The measure of the intragroup variation, ${S}_{p}^{2}$, was calculated to be 0.82 using Eq. (6). The measure of intergroups variation, $({nS}_{m}^{2}),$ was calculated to be much higher: 180.12, using $n$ times the result of Eq. (9).

These two measures were consolidated using the weights of ${w}_{1}=\mathrm{0,93}$ and ${w}_{2}=\mathrm{0,07}$ respectively, calculated using Eq. (12) and Eq. (13). The weighted average resulted in ${S}_{a}^{2}=13.19$. The square root of ${S}_{a}^{2}$ is our estimate for the standard deviation of the emission factor: ${S}_{a}=3.6$.

Notice that ${S}_{a}$ is bigger than each of the samples’ standard deviation. This is not surprising since the three samples presented means that are, comparably, quite apart from each other. In conclusion, we estimate the emission factor for the rice produced in the region of Huê in central Vietnam to be 25.6 ± 3.6 kg of methane per ton of rice produced. The application of the method was straightforward and simple to justify and interpret.

The case study revealed a series of issues that must be considered in practical applications:

(A) If we take samples from field sites that differ in terms of edaphic or climate characteristics or if we take samples from different years or seasons, the discrepancy in the emissions data would be incompatible with the hypothesis that the data come from the same population and the resulting standard deviation might be big and meaningless.
(B) Data in practical cases might contain outliers. Failing to detect and correct for these would distort the results significantly.
(C) The criterion of weighting sample averages using the inverse of their variances might produce severe outbalances, especially if some data of sample variances are close to zero.

## Discussion

In order to establish a confidence interval estimator for the emission factor *F*, it is necessary to estimate the variance of* F* and the probability distribution of the estimator. We have studied three methods to estimate the variance of* F*: 1) the pooled variance $\left({S}_{p}^{2}\right)$; 2) the method of the means ${(nS}_{m}^{2})$ and 3) the ANOVA based method $\left({S}_{a}^{2}\right)$.

The first method, ${S}_{p}^{2}$, combines information on the variances contained in each available sample but does not consider the possible distinctions between the expected values of these samples. This situation is applicable to cases where the mean of* F* can be estimated by other methods (for example, a linear regression where an explanatory variable assumes different values in each sample) but the variances within each sample are all the same (although unknown).

The second method, ${(nS}_{m}^{2})$, is applicable in situations where we do not have information about the internal variations of each sample and only know the averages of each sample. Apparently, it may seem to be a particular case of the previous situation. However, it is necessary to use a different approach and consider that each sample average is an element, and the average of these elements is an estimator of *μ*. The sample variance between these elements, ${S}_{m}^{2}$, however, is not an estimator of *σ*^2^, the variance of* F*, but rather the estimator of the variance of the “*F* averages”. Assuming, for example, that each sample mean is the result of the average of *n* elements, ${S}_{m}^{2}$ is an estimate of *σ*^2^/*n*. Thus, unless we know the value of $n$, the number of elements in each sample, we cannot use ${S}_{m}^{2}$ to determine an estimator of *σ*^2^ and, consequently, cannot determine a confidence interval comparable to the previous ones. Using ${S}_{m}^{2},$ one could determine a confidence interval for *μ* but we must be careful to interpret it properly: it is the confidence interval for the averages of $n$ values of* F*.

The third method for estimating the variance of emission factors may be considered original: the proposed ${S}_{a}^{2}$ is based on the law of total variance; it captures within groups variation as well as intergroups variation; it is easy to compute, and it has convenient statistical properties.

Showing that the distribution of ${S}_{a}^{2}$ is scaled chi-square, in the usual context of “independence, normality and homogeneity” was crucial to determine the confidence interval for the expected value of* F*. The qualities of the estimators for *σ*^2^ were examined theoretically and using simulation. The simulation served to confirm their properties and to illustrate their different applicability contexts.

The theoretical developments and simulations presented in this document have shown that estimating the variance of* F* using ANOVA principles produces an estimator of *σ*^2^ that is unbiased and of minimum variance among the ones examined. In different contexts, Swallow and Monahan [37] have also argued in favor of ANOVA estimators of variance. In the context of estimating the emission of GHG based on a pool of estimates presented in the accredited literature, we do not know of any other method that surpasses the qualities of ${S}_{a}^{2}$.

Statistically, ${S}_{a}^{2}$ is unbiased, has small variance and has a known probability distribution and from a practical point of view, its computation requires information that is usually available and, finally, it does not neglect potentially important information (${S}_{p}^{2}$ neglects the intergroups variability and ${(nS}_{m}^{2})$ neglects the intragroup variability).

## Conclusions

Using pooling of estimates, an efficient point estimator for *µ*, the expected value of the emission factor of an activity, *F*, relies on a weighted average. The weights, however, are not obvious if we do not know, and have to estimate, the variances involved. Therefore, we presented suggestions, based on meta-analysis theory, to form point estimators for *µ* and have studied three methods to estimate *σ*^2^, the variance of* F.*

One contribution of this work resides in the discussion of situations where each estimator of *σ*^2^, namely: $${S}_{p}^{2}, ($$ n $${S}_{m}^{2})$$ and $${S}_{a}^{2}$$, is best suited for. The choice depends on data availability and the characteristics of the original population from where the data is taken.

The first two estimators are known from the literature and the third might be considered a contribution of this work. The estimator ${S}_{a}^{2}$ is derived from the ANOVA theory. We have demonstrated its properties, including its distribution as a scaled chi-square random variable, and have indicated different possibilities for its computation. We have also shown that ${S}_{a}^{2}$ is unbiased and is the most precise estimator for the variance of* F* under the assumption that *F* is distributed as a random variable with fixed, though unknown, expected value and variance.

Therefore, we have shown how to use a combination of reported emission factors to form the narrowest confidence interval for the true emission factor of interest, thus improving the reliability and accuracy of GHG emission estimates.

The procedure was applied to the case of CH_4_ emissions from rice plantations in Central Vietnam. Available databases suggested three possible emission factors ranging from 20.7 ± 0.8 to 28.6 ± 0.28 kg/ton. After applying the suggested procedure, the emission factor was estimated to be 25.6 ± 3.6 kg of methane per ton of rice produced. Other procedures would have resulted in less precise or in biased estimators.

### Supplementary Information

**Additional file 1.** Mathematical foundations

## Data Availability

Additional file Information of the online version contains Additional file materials.

## Abbreviations

*F*: Population of possible values for the emission factor of a certain activity or product. The observations of this variable *F* are ${f}_{ij}.$
*i*: One of the k groups of observations. Each group represents a sample set of observations
*j*: Index for one observation in the ${i}^{th}$ sample.
${n}_{i}$: The number of elements in the ${i}^{th}$ sample.
${\underline{{f_{i} }} }$: The average of the elements in the ${i}^{th}$ sample ($\frac{1}{{n}_{i}}{\sum }_{j=1}^{{n}_{i}}{f}_{ij})$
*N*: Total number of elements $\left({\sum }_{i=1}^{k}{n}_{i}\right)$
*µ*: The true, but unknown, expected value, also called mean, of *F*
*σ*^2^: The true, but unknown, variance of *F*

## Footnotes

1 S_i_^2^ is a scaled chi-square random variable when ${C}_{i}^{2}=\frac{\left({n}_{i}-1\right){S}_{i}^{2}}{{\sigma }^{2}}$ is a chi-square random variable.

2 Observe that ${C}_{m}^{2}=\frac{(k-1){S}_{\boldsymbol{m}}^{2}}{{(\sigma }^{2}/n)}$ is a chi-square random variable with ${df}_{m}$ = (k-1) degrees of freedom.
