# Supplementary figures and images for: Procedures to combine estimators of greenhouse gases emission factors
Source: Carbon Balance Manag. 2024 Feb 5;19:4. doi: 10.1186/s13021-024-00250-8 (PMC11342513; doi:10.1186/s13021-024-00250-8)

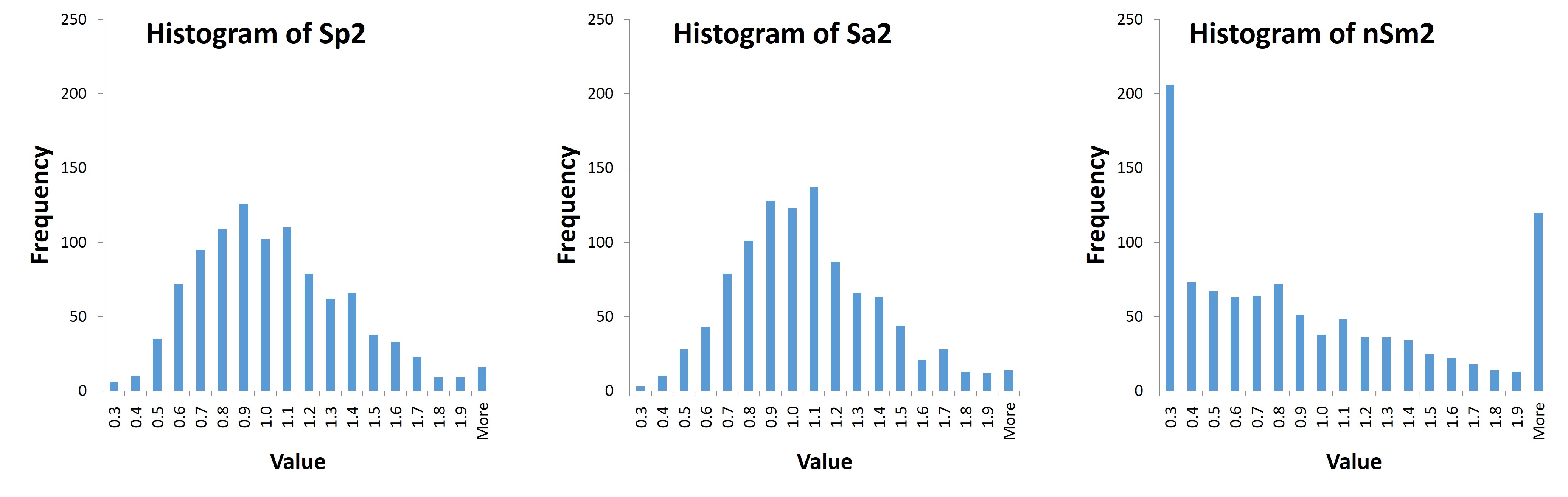

Supplement: Supplementary file 1 — Additional file 1. Mathematical foundations [file 13021_2024_250_MOESM1_ESM.jpg]
